# 3D comparison of dental arch stability in patients with and without cleft lip and palate after orthodontic/rehabilitative treatment

**DOI:** 10.1590/1678-7757-2018-0434

**Published:** 2019-06-13

**Authors:** Jorge Tomasio CABALLERO, Maria Giulia Rezende PUCCIARELLI, Victor Fabrizio Cabrera PAZMIÑO, Victor Prado CURVÊLLO, Márcio de MENEZES, Chiarella SFORZA, Simone SOARES

**Affiliations:** 1 Universidade de São Paulo, Faculdade de Odontologia de Bauru, Bauru, São Paulo, Brasil.; 2 Universidade de São Paulo, Hospital de Reabilitação de Anomalias Craniofaciais, Bauru, São Paulo, Brasil.; 3 Universidade do Estado do Amazonas, Amazonas, Manaus, Brasil.; 4 Università degli Studi di Milano, Facoltà di Medicina e Chirurgia, Dipartimento di Scienze Biomediche per la Salute, Functional Anatomy Research Center (FARC), Milan, Italy.; 5 Universidade de São Paulo, Hospital de Reabilitação de Anomalias Craniofaciais, Departamento de Prótese e Periodontia, Bauru, São Paulo, Brasil.

**Keywords:** Cleft lip, Cleft palate, Dental models, Dental arch, Three-dimensional imaging, Rehabilitation

## Abstract

This study aimed to compare the linear dimensions of the dental arches of adult patients with complete unilateral cleft lip and palate (UCLP) after orthodontic and prosthetic treatment with fixed partial dentures (FPD) to patients without clefts, using 3D technology. This retrospective longitudinal study sample consisted of 35 subjects divided into two groups. Included in this sample were 15 complete UCLP individuals who had received orthodontic treatment before rehabilitation with a fixed partial denture (FG), as well as 20 patients without cleft as control group (CG). All patients were aged between 18 and 30 years. Digital dental casts were obtained in two stages: (T1) end of orthodontic treatment and (T2) one year after prosthetic rehabilitation (FG); and (T1) end of orthodontic treatment and (T2) one year after removal of the orthodontic appliance (CG). Intercanine, interfirst premolar and intermolar distances, and incisor-molar length were obtained. A precalibrated and trained examiner performed the assessments. Intergroup differences between T2 and T1 were compared between the groups using the t test or Mann-Whitney test with a significance level of 5% (p<0.05). The intercanine distance variation (T2-T1) showed statistical difference (p=0.005) increasing in the FG group and decreasing in the CG group. In the interfirst premolar distance variation, FG decreased, while CG increased with statistically significant difference (p=0.008). The intercanine distance of individuals with cleft showed stability, while that of the CG had no stability. The CG showed stability in the interfirst premolar distance, while FG had no stability. These findings showed that the FPD is capable of restricting orthodontic results, leading to a stabilization of the dental arches.

## Introduction

Cleft lip and palate (CLP) is the most prevalent congenital malformation (1 in every 500 to 700 births *per* year) and is considered a public health burden according to the World Health Organization.^1^ Oral clefts may involve the lip, alveolus and palate, and occur up to the 12^th^ week of intrauterine life.^1^ An early diagnosis may occur during pregnancy after ultrasound examination,^2^ but rehabilitative treatment starts immediately after birth with primary surgeries generally being performed up to the age of 12 months. Although primary surgeries correct aesthetics and function, they can have deleterious effects on maxillary growth.^3^ ^-^ ^8^

For the success of rehabilitative treatment, study model dental casts should be obtained for diagnosis, planning and monitoring morphological information. These become part of the patient’s dental record, which should be systematically maintained from birth through all phases of treatment^9^ to enable the longitudinal evaluation of rehabilitative treatment.^10^ Despite the valuable information obtained with study casts, comparative studies must deal with the inconvenience of transporting the casts. Such challenges have led to alternative methods for morphological evaluation of anatomic structures. Thus, the three-dimensional (3D) analysis of the dental arches is a significant shift in data collection,^10^ ^-^ ^15^ showing several advantages.^11^ ^,^ ^12^ ^,^ ^15^ ^-^ ^18^ Studies comparing measurements on digital dental images and on study dental casts concluded that 3D images are clinically acceptable and reproducible.^16^ ^-^ ^18^

Professionals must be aware of dimensional changes in the dental arches of individuals with cleft lip and palate because these alterations influence the outcomes of the rehabilitative process,^19^ which aims not only to anatomically and functionally rehabilitate, but also to restablish the social acceptance of the individual.^20^

Studies on the evaluation of the dental arch dimensions of individuals with CLP after orthodontics and on the stability achieved after prosthetic treatment are lacking. Therefore, this study aimed to compare the linear dimensions of the dental arches of patients with complete unilateral cleft lip and palate (UCLP) after orthodontic and prosthetic treatment with a fixed partial denture with the dimensions of patients without cleft lip and palate immediately after orthodontics and one year after removal of the orthodontic appliance. The hypothesis was that no stability of the dental arches would be observed in non-cleft patients after orthodontic treatment, as well as for cleft patients after orthodontic and prosthetic treatment. The information provided will aid in a better understanding of the factors interfering in the stability of the dental arches of individuals with complete UCLP, mainly the definitive outcome of rehabilitative treatment.

## Material and methods

### Sample selection

This study was submitted to and approved by the Institutional Review Board of the Hospital for the Rehabilitation of Craniofacial Anomalies (HRAC/USP) under protocol CAAE #50808215.2.0000.5441. All participants were selected from the files of HRAC/USP and the Bauru School of Dentistry (FOB/USP).

For all patients from both institutions, three-dimensional digital images of the dental casts were obtained. The individuals with and without complete UCLP, both genders, were aged from 18 to 30 years. The inclusion criteria were as follows: individuals with and without complete UCLP with anterior and/or posterior crossbite, with all dental casts at the evaluated periods. The exclusion criteria comprised individuals with associated syndromes or malformations, those submitted to orthognathic surgery, those who underwent premolar extraction, and those who wore overdentures, a complete denture, or implant-supported fixed complete denture. Ninety-seven dental casts were evaluated; 62 did not meet the inclusion criteria and were excluded because they lacked casts at all evaluated phases.

Based on a pilot study, sample size calculation showed that to detect a minimum difference in the transversal measurement of 0.8 mm, with a standard deviation of 0.7 mm, a level of significance of 5%, and a test power of 80%, 15 individuals *per* group were necessary. Thus, 35 individuals were divided into two groups:

Control group (CG) – 20 non-cleft patients who had undergone previous orthodontic treatment (9 male and 11 female, mean age/years 22.4±4.65).

Group Fixed Partial Denture (FG) – 15 individuals with complete UCLP who had received orthodontic treatment before rehabilitation with a fixed partial denture of three elements (7 male and 8 female, mean age/years 26.6±٣.٧٧).

All patients (CG and FG) received similar orthodontic treatment, and rapid maxillary expansion to correct and align the maxillary arch. When the orthodontic appliance was removed, the patient wore a Hawley appliance while waiting for prosthetic treatment. The evaluation was performed on 3D images of the maxillary dental cast obtained at the following time points:

*Control Group (CG):*

- After orthodontic treatment (T1)

- One year after the end of orthodontic treatment (T2)

*Group Fixed Partial Denture (FG):*

- After orthodontic treatment, with prosthetic requirements (T1)

- One year after prosthetic rehabilitation (T2)

### Digitation of casts

The dental casts obtained from the files of both institutions were digitized with a laser scanner (3Shape’s R700TM Scanner, Copenhagen, Copenhagen, Denmark) and analyzed with Appliance Designer Software (3Shape, Copenhagen, Copenhagen, Denmark).

### Obtaining measurements

**Figure 1:**
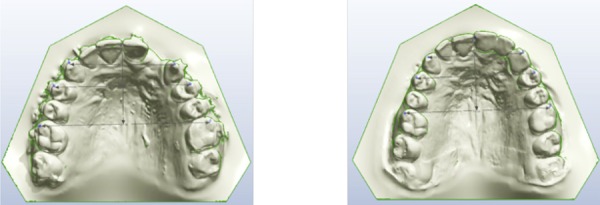
Landmarks used for the analysis of the digital images for group FG before and after fixed partial denture placement

**Figure 2:**
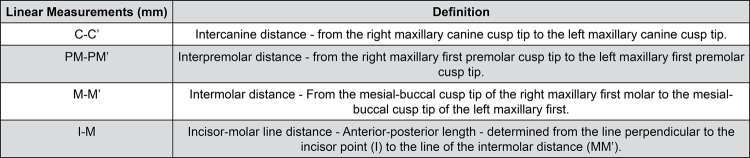
Definition and description of the Linear Measurements (mm)

A set of landmarks was identified on the 3D images of dental arches to obtain the linear measurements (Figures 1 and 2). All measurements were performed point-by-point with the software tool: intercanine distance,^4^ ^,^ ^21^ interfirst premolar distance,^8^ intermolar distance, and total length of the dental arch from the incisor to the molar line.^21^ ^,^ ^22^ The variation of the distances between the study time points was obtained by the difference between values at T2 and values at T1 (Δ). This difference was considered for the statistical analysis of dental arch stability.

### Statistical analysis

All statistical tests were performed with statistical software (Statistica for Windows - Version 7.0 – StatSoft, TIBCO Software, Palo Alto, CA, USA). To analyze the intra-rater error, the sample was measured again 15 days after the first evaluation. To calculate the systematic error, the paired *t* test was used. The random error was determined by using the Dahlberg formula.^23^

The Shapiro-Wilk test was applied to verify data normality. Accordingly, the *t* test was used to test differences during the analyzed time points in dental arch measurements with a normal distribution, while the Mann-Whitney was used for those with a non-normal distribution. All tests were set at a 5% level of significance. Mean and standard deviation were reported for normally distributed data, while median and interquartile range were reported for not-normally distributed data.

## Results

### Error of method

The intra-rater reproducibility was verified with the paired *t* test and the Dahlberg formula between the measurements performed by the same examiner (JTC) within the 15-day interval.

### Maxillary dimensions

Tables 1 and 2 show the inter group comparisons for each period (T1 and T2) of the evaluated groups and observe the main alterations in the studied periods. At T1, PMPM’ and MM’ distances were similar, while CC’ and IM’ were significantly larger in the CG (Table 1). At T2, only IM showed statistical difference, being larger in the CG (Table 2).

**Table 1:** Analysis of the linear dimension means (mm) in the studied groups after orthodontic finalization (T1)

	CG		FG		
T1	Mean (median)	SD (ID, 25%/75%)	Mean (median)	SD (ID, 25%/75%)	P
CC'	35.17	± 2.19	31.85	± 3.87	**0.003** ^*t^
PMPM'	43.31	± 2.80	43.01	± 2.71	0.75^t^
MM'	(52.83)	(51.46 – 55.08)	(52.33)	(49.35 – 54.74)	0.278^α^
IM'	(27.80)	(26.87 – 29.55)	(24.92)	(20.99 – 27.40)	**0.003** ^*α^

SD - standard deviation

ID - interquartile deviation

independent t test

^α^ Mann-Whitney test (nonparametric)

^*^ statistically significant difference (p<0.05)

**Table 2:** Analysis of the linear dimension means (mm) in the studied groups after the end of orthodontic/rehabilitative treatment (T2)

	CG		FG		
T2	Mean (median)	SD (ID, 25%/75%)	Mean (median)	SD (ID, 25%/75%)	P
CC'	34.91	± 2.34	33.36	± 3.14	0.10^t^
PMPM'	43.59	± 2.61	42.15	± 2.64	0.12^t^
MM'	52.82	± 2.62	51.17	± 2.87	0.09^t^
IM'	28.87	± 2.21	24.64	± 3.83	**0.0003** ^*t^

SD - standard deviation

ID - interquartile deviation

independent t test

α Mann-Whitney test (nonparametric)

* statistically significant difference (p<0.05)

The measurement change (T2-T1) showed a negative ΔC for the intercanine measurement in the CG, while FG had a positive ΔC, indicating an increase in this transversal distance, with statistical significance differences between groups (Table 3). A different behavior occurred for the change in the interfirst premolar distance (PMPM’): negative ΔPM for FG (-0.86±0.97) and positive ΔPM for CG (0.28±1.331 mm), with statistically significant differences between groups (p=0.008), indicating a reduction of the linear measurement for the group with cleft (Table 3). Both groups showed a reduction in the intermolar distances (MM’), without statistically significant differences between groups (p>0.05) (Table 3). Both groups showed an increase in the incisor-molar length (IM), without statistically significant differences between groups (p=0.375) (Table 3).

**Table 3:** Comparison of the intergroup differences (T2-T1=Δ)

	CG		FG		
T2-T1	Mean (median)	SD (ID, 25%/75%)	Mean (median)	SD (ID, 25%/75%)	P
CC'	(-0.25)	(-1.1/0.4)	(0.7)	(-0.2/2.6)	**0.005** ^*α^
PMPM'	0.28	±1.31	-0.86	±0.97	**0.008** ^*t^
MM'	(-0.45)	(-1/0.2)	(-0.3)	(-1/0.1)	0.854^α^
IM'	0.46	±1.03	0.13	±1.09	0.375^t^

SD - standard deviation

ID - interquartile deviation

independent t test

^α^ Mann-Whitney test (nonparametric)

^*^ statistically significant difference (p<0.05)

## Discussion

The Class III malocclusion pattern is the most common in individuals with CLP because primary surgeries can cause scar tension. This prevents anterior-posterior and transverse maxillary expansion and may account for the discrepancy in the maxillo-mandibular relationship. The goal of orthodontics is to achieve maxillary expansion and stabilize the segmented arch by means of a secondary alveolar graft^10^ ^,^ ^24^ to allow orthodontic movement.

However, it is not always possible, after orthodontics, to establish a satisfactory occlusal and aesthetic relationship, since the complete UCLP patient has no lateral incisor. In some patients, orthodontic treatment replaces the lateral incisor with the canine to prevent the need for either a fixed partial denture or implant-supported prosthesis. Indeed, implant placement in the cleft area may be contraindicated because of poor bone/gingival tissue, quality and quantity. Based on this condition of individuals with complete UCLP, this study aimed to verify whether after the fixed partial denture placement in the cleft area, the stability, obtained with the orthodontic treatment, was maintained. The hypothesis studied was partially rejected, because the difference between T2 and T1 showed stability in the FG, in CC’ and IM length, and in the CG in PMPM’ and IM length, and there was no stability in the FG at the distances PMPM’ and MM’ and in the CG at CC’ and MM’.

In this study, the linear distances between the canines in CG and GF in T1 were significantly different, being shorter in GF relative to CG (p=0.003). As orthodontics does not always restore the canine to its original position in the dental arch, taking it to the lateral incisor position instead, it is possible to have different measures between groups in T1 (Table 1). Throughout the linear measurements, we were sometimes able to observe the canine in the lateral incisor area, and this condition may have led to the observed statistical alterations. This bias could be eliminated by excluding all patients who presented the canine out of its correct position in the dental arch.

At T2, the intercanine distances of the CG decreased and those of the FG increased (Table 2). The difference (ΔC) also showed the same behavior (Table 3). This highlights the stability of the canines in the individuals with complete UCLP, a result different from that obtained by Li and Lin^25^ (2007), who found a relapse, especially in the upper canine and first premolar region; however, most of the treatment effect on the upper arch remained after retention.

The intragroup comparisons of the interfirst premolar distances at both periods did not show statistically significant difference. The ΔPM showed statistically significant differences between groups because of the decrease in the PMPM’ of group FG. This fact leads us to infer that the prosthesis installed in the lateral incisor area maintains the stability achieved by the orthodontic treatment.

No statistically significant differences occurred in the intermolar distance at either time points (p>0.05). The same behavior occurred for the ΔM comparison between the groups. Both groups showed a reduction in ΔM, that is, lack of stability. This result supported the importance of the FPD in stabilizing the results obtained by the orthodontic treatment.

It is important to emphasize that the literature reports few studies on the stability of dental arches of individuals with complete UCLP at the end of orthodontic/rehabilitative treatment with fixed prostheses.^8^ ^,^ ^26^ ^,^ ^27^ Brägger, Burger, and Ingervall^26^ (1991) evaluated the stability of dental arches of individuals with complete UCLP over eight years and observed a reduction in maxillary width and interfirst premolar and intermolar distances, a result similar to that of this study. By following individuals with complete UCLP for 13.5 years, Ramstad and Jendal^27^ (1997) observed similar results, that is, decreasing interfirst premolar and intermolar distances. However, these authors found an increase in intercanine distances, unlike this present study. Ramstad and Jendal^27^ (1997) also affirmed that most of the posttreatment dental changes occurred in the first five years and that complete stability was not reached, even at the final period. Marcusson and Paulin^8^ (2004) analyzed transversal distance in individuals with complete UCLP who received a fixed partial denture with a mean follow-up period of 5.6 years and found a decrease and significant deterioration in the maxillary arch distances, irrespective of the type of retention (no retention, bonded retainer, and onlay/fixed bridge).

The comparison between individuals with and without clefts aimed to verify whether cleft treatment outcome is similar to the outcome obtained in individuals without clefts, because the main goal is to reintroduce complete UCLP patients into society.

The analysis of the IM length, which assesses the anterior-posterior arch length, showed the alterations caused by the primary surgeries. At T1, the FG had a lower value than the CG, with statistically significant differences between groups. The studies of Athanasiou, Mazaheri and Zarrinnia^28^ (1986) and Ayub et al.^24^ (2016) verified that the arch length of individuals with complete UCLP is shorter than that of individuals without clefts in both the primary and permanent dentitions, corroborating with the findings of the present study. The change in the IM length (ΔIM) between groups showed no statistically significant differences. The rationale behind this is that the reduction had already occurred during childhood and was perpetuated in adolescence, not allowing for compensation in maxillary growth, which was contained by the primary surgeries. We highlight that, in this study, we excluded individuals who had undergone orthognathic surgery. Thus, regarding transverse dimensions, orthodontic treatment in subjects with cleft seems to be much more unstable than in subjects without cleft.

As limitations of this study, the dimension of width or length of the original defect of each patient was not established, surgeons involved and degree of orthodontic expansion, in the beginning of the treatment, were not evaluated. With regard to cleft dimensions, there is no available classification if the defect is severe or moderate. Orthodontic expansion is performed, as much as possible, to uncross the bite.

Individuals with complete UCLP undergo longer treatments, and the rehabilitative treatment only begins after the orthodontic treatment. The maxillary arch of these individuals may have some dimensional alterations that can change the final outcomes. In addition to maintaining the CC width, the loss of premolar distance is evidence that if the prosthesis is not inserted, this being a rehabilitation issue *per se*, the patient can quickly lose transverse dimension. Professionals must be aware that treatment is not finished with the fixed partial denture installation. Periodic follow-up appointments are necessary and should include occlusion assessments, since dental alterations may occur over time. In the long term, alterations to the dental arches may not significantly alter the aesthetic outcome, but they may directly influence the necessity of occlusal adjustments due to the lack of dental arch stability. This would explain the higher demand for occlusal adjustments in individuals with UCLP after prosthetic treatment.

The literature lacks studies on the stability of the dental arches of adults with oral clefts. Also, different rehabilitative centers have different treatment protocols. Thus, further studies are necessary to understand the stability of the maxillary arch in individuals with UCLP at the end of rehabilitative treatment.

## Conclusion

The intercanine distance was stable in the FG and unstable in the CG, showing that the fixed partial denture is capable of restraining orthodontic outcomes;

The interfirst premolar distance was unstable in the fixed partial denture group and stable in the control group;

The intermolar distances values of both groups showed reduction after treatment;

The incisor-molar line length was stable for both groups because the maxillo-mandibular discrepancy is maintained from childhood to adulthood in individuals with cleft lip and palate.

The present findings showed that the FPD is capable of containing orthodontic results, leading to a stabilization of the dental arches.
